# The MTH1 inhibitor TH588 is a microtubule-modulating agent that eliminates cancer cells by activating the mitotic surveillance pathway

**DOI:** 10.1038/s41598-019-51205-w

**Published:** 2019-10-11

**Authors:** Nadia Gul, Joakim Karlsson, Carolina Tängemo, Sanna Linsefors, Samuel Tuyizere, Rosie Perkins, Chandu Ala, Zhiyuan Zou, Erik Larsson, Martin O. Bergö, Per Lindahl

**Affiliations:** 1 Wallenberg Laboratory, Department of Molecular and Clinical Medicine, Institute of Medicine, University of Gothenburg, SE-413 45 Gothenburg, Sweden; 2 Department of Surgery, Institute of Clinical Sciences, University of Gothenburg, SE-416 85 Gothenburg, Sweden; 3 Department of Medical Biochemistry and Cell Biology, Institute of Biomedicine, University of Gothenburg, SE-405 30 Gothenburg, Sweden; 4 Centre for Cellular Imaging, University of Gothenburg, Box 413, SE-40530 Gothenburg, Sweden; 5 Department of Biosciences and Nutrition, Karolinska Institutet, SE-141 83 Huddinge, Sweden

**Keywords:** Target validation, Targeted therapies

## Abstract

The mut-T homolog-1 (MTH1) inhibitor TH588 has shown promise in preclinical cancer studies but its targeting specificity has been questioned. Alternative mechanisms for the anti-cancer effects of TH588 have been suggested but the question remains unresolved. Here, we performed an unbiased CRISPR screen on human lung cancer cells to identify potential mechanisms behind the cytotoxic effect of TH588. The screen identified pathways and complexes involved in mitotic spindle regulation. Using immunofluorescence and live cell imaging, we showed that TH588 rapidly reduced microtubule plus-end mobility, disrupted mitotic spindles, and prolonged mitosis in a concentration-dependent but MTH1-independent manner. These effects activated a USP28-p53 pathway – the mitotic surveillance pathway – that blocked cell cycle reentry after prolonged mitosis; USP28 acted upstream of p53 to arrest TH588-treated cells in the G1-phase of the cell cycle. We conclude that TH588 is a microtubule-modulating agent that activates the mitotic surveillance pathway and thus prevents cancer cells from re-entering the cell cycle.

## Introduction

The mut-T homolog 1 (MTH1, also known as NUDT1) inhibitor TH588 has shown promise as an anticancer compound in preclinical studies^1,2^. It is toxic to a wide range of human cancer cell lines at concentrations that are tolerated by primary or immortalized cells^1^. In addition, daily injections of TH588 in mice markedly reduced the growth rate of a patient-derived visceral metastasis of a malignant melanoma at concentrations that did not affect body weight, blood cell counts, or liver/heart/kidney parameters^1^. The cytotoxic effect of TH588 on human cancer cell lines has been confirmed in other laboratories^3–7^, supporting further development of this compound.

Although independent studies have confirmed that TH588 inhibits MTH1 at nanomolar concentrations^4–8^, accumulating evidence suggests that its anti-cancer effect is mediated by other mechanisms: First, the concentrations of TH588 required for its cytotoxic effect are several fold higher than those required for MTH1 inhibition^1,5^. Second, a number of alternative MTH1 inhibitors have been developed, none of which are toxic when tested on human cancer cell lines^4–7^. Third, CRISPR-mediated knockout of MTH1 failed to reproduce the cytotoxic effect of MTH1 knockdown that was previously reported in human cancer cell lines^5,9^. And fourth, mice lacking MTH1 show a small but significant increase in spontaneous cancers^10^. A number of alternative mechanisms have been suggested for the anti-cancer effects of TH588 including lipophilicity-related effects, isoform-specific MTH1 targeting, tubulin depolymerization, oxidative damage, and downregulation of the PI3K-Akt-mTOR axis^5,6,11–14^, but the question remains unresolved^12,15^. There are no reports on the use of unbiased screens to define genes and pathways that underlie TH588’s effects.

Here we performed a pooled lentiviral CRISPR screen to identify gene knockouts that rescue H460 human lung cancer cells from the cytotoxic effect of TH588. Data from such screens can identify mechanisms behind drug effects and drug resistance mechanisms.

## Results

### CRISPR screen of TH588-treated cells identified complexes and pathways associated with mitotic spindle regulation

**Figure 1:**
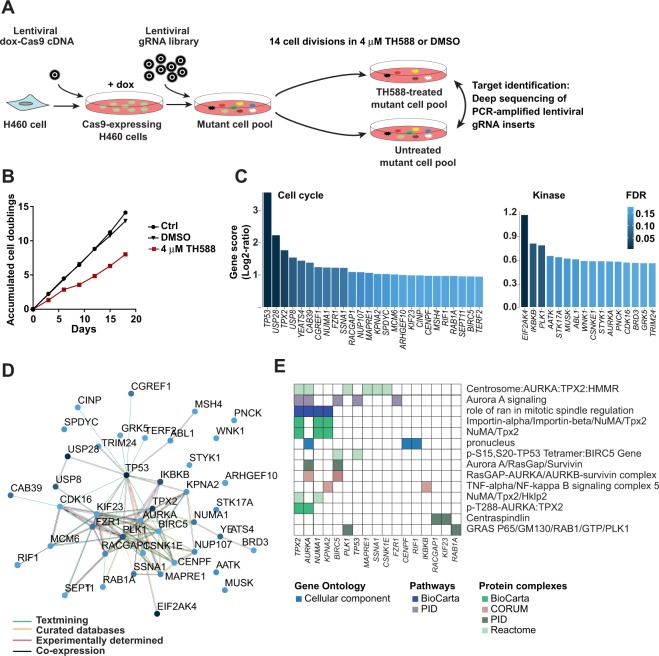
CRISPR/Cas9 screening of TH588-treated cells identified protein complexes and pathways associated with mitotic spindle regulation. (**A**) Doxycycline-inducible Cas9-expressing cells were infected with lentiviral gRNA libraries to generate complex mutant cell pools (MCPs) for screening. The MCPs were passaged in TH588 or DMSO for 14 cell divisions before determining the gRNA repertoire (and hence the repertoire of mutations) in the selected cell populations by massive parallel sequencing of PCR-amplified lentiviral inserts. (**B**) Growth curves showing accumulated cell doublings of MCPs that were passaged in TH588 or DMSO. (**C**) Gene scores for cell cycle genes (left) and kinase genes (right), analogous to average gRNA fold-change (Log2-ratio) in TH588-treated MCPs compared to controls as calculated with the MAGeCK MLE algorithm. Genes with false discovery rates (FDR) < 0.2 are shown. (**D**) A protein interaction network constructed with candidate genes for both libraries (FDR < 0.2) using the STRING database of known or predicted protein-protein interactions. The STRING database integrates diverse types of evidence and the color of the edges corresponds to the type of supporting evidence. The color of the nodes corresponds to the FDR value presented in panel C. (**E**) Graphic representation of candidate genes and their corresponding functional annotations for gene ontology terms and pathways and protein complexes that were statistically overrepresented among candidate genes with FDR < 0.1 in our screen. The analysis was performed with ConcensusPathDB and shows annotations with *q* < 0.05 in the respective database.

To elucidate mechanisms behind the pharmacological anti-cancer effect of TH588, we performed pooled lentiviral CRISPR screens to identify gene knockouts that rescue proliferation or viability of TH588-treated human H460 lung cancer cells (Fig. 1A). To make H460 cells accessible for screening, we infected cells with lentiviral *Cas9* and generated clones expressing doxycycline-inducible Cas9 (Supplementary Fig. S1A). Cas9-expressing cells were infected with two guide RNA (gRNA) libraries targeting 1000 cell cycle genes and 500 kinase genes, and treated with blasticidin to produce mutant cell pools^16^. Each gene was targeted by 10 different gRNAs. Massive parallel sequencing of PCR-amplified lentiviral inserts showed that 9 or 10 gRNAs per gene were detected for more than 95% of the targeted genes, indicating that virus transduction efficiency and sequencing depth were sufficient (Supplementary Fig. S1B).

To select for mutations that rescue the cytotoxic effect, we passaged the mutant cell pools for 18 days in 4 µM TH588, which markedly reduced cell proliferation (Fig. 1B). In a parallel study, we passaged the same mutant cell pools in 2 µM auranofin, a thioredoxin reductase inhibitor that kills cells by inducing oxidative stress. Control mutant cell pools were passaged with and without DMSO. The abundance of all gRNAs was determined by massive parallel sequencing of PCR-amplified lentiviral inserts. Principal component analyses showed that TH588- and auranofin-treated samples separated along the two first principal components, whereas untreated and DMSO-treated control samples clustered together (Supplementary Fig. S1C). These results indicate that both drugs induced gRNA selection, and that the mechanisms leading to enrichment or depletion of gRNAs in these cell populations were distinct.

Gene scores were calculated for each gene based on the average gRNA fold-change in TH588-treated mutant cell pools compared to controls (Supplementary Data 1). Statistical analysis identified 42 candidate genes whose gRNAs were enriched in TH588-treated samples at 20% false discovery rates (FDR < 0.2, Fig. 1C). A protein interaction network constructed with candidate genes for both libraries using the STRING database of known or predicted protein-protein interactions revealed a densely connected network with *TP53*, *USP28*, *TPX2*, *NUMA1*,* PLK1*, and *AURKA* as central components (Fig. 1D), in agreement with their high positions in the ranked gene lists (Fig. 1C). An overrepresentation analysis of functional interaction networks with ConsensusPathDB further supported functional associations between the top-ranked genes (Supplementary Data 2). A highly dominating theme was pathways and protein complexes involved in mitotic spindle regulation (Fig. 1E).

### TH588 is a microtubule-modulating agent

**Figure 2:**
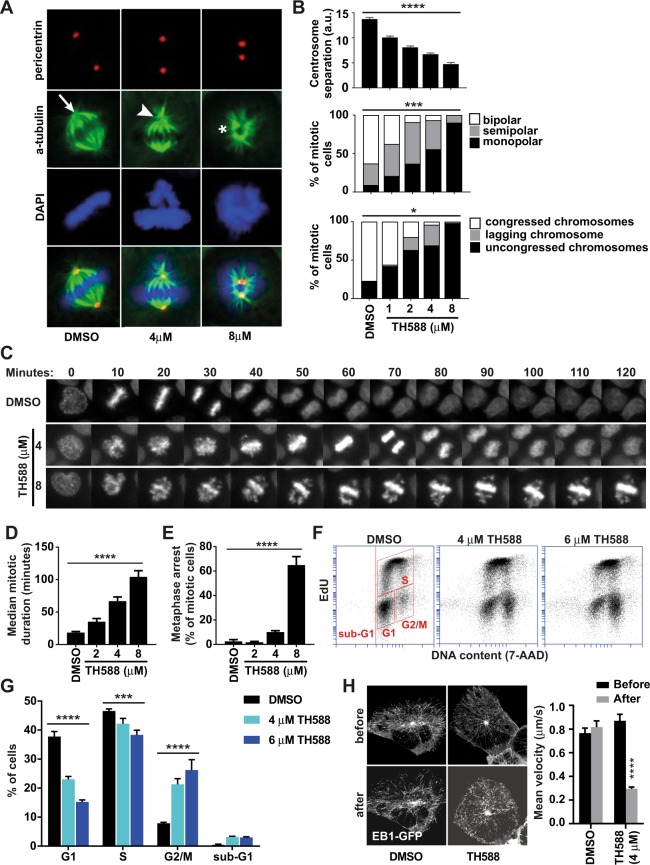
TH588 is a microtubule-modulating agent. (**A**,**B**) Photomicrographs of unsynchronized mitotic cells treated with DMSO or TH588 for 2 hours showing pericentrin (red), α-tubulin (green), and chromatin (blue, DAPI). Graphs showing centrosome separation (top panel), percentage of mitotic cells with bipolar (arrow) or semipolar (arrowhead) or monopolar (asterisk) spindles (middle panel), and percentage of mitotic cells with congressed, lagging, or uncongressed chromosomes (bottom panel) (n = 3 replicates/concentration, 100 mitoses/replicate). (**C**) DNA of live cells that were stained with Hoechst sir-DNA for time-lapse observation of chromosome congression and segregation in the presence of DMSO or TH588. (**D**,**E**) Quantification of time-lapse data showing (**D**) mitotic duration (n = 3 replicates/concentration, median of 57–152 mitoses/replicate) and (**E**) percentage of mitotic cells that were permanently arrested in metaphase (n = 4 replicates/concentration, 33–148 mitoses/replicate). (**F**) Plots showing DNA content (7-AAD) and EdU-incorporation of cells after 24-hour incubation with DMSO or TH588 and 2-hour incubation with EdU. (**G**) Graphs showing cell cycle distribution of cells in (F). The gating strategy is indicated by inserted squares (n = 3 replicates/concentration). (**H**) Kymographs (walking average) of representative EB1-GFP-expressing H460 cell before (above) and after (below) administration of DMSO or TH588. Graph shows plus-end velocity before and after administration of DMSO or TH588 (n = 8 cells, mean of 10 tracks/cell). The statistics used were linear regression with test of non-zero slope (**B**,**D**,**E**), two-way ANOVA, simple effects within rows (**G**), and paired student’s t-test (**H**). *P* values in panel (B,D,E,H) and *q* values in panel (G). ^*^P < 0.05, ^***^P or *q* < 0.001, ****P or *q* < 0.0001. Graphs show mean +/− SEM.

Mitotic spindle assembly is a process involving centrosomes and microtubules. Centrosomes duplicate during the S phase of the cell cycle, migrate to opposite cell poles during the prophase of mitosis, and organize bipolar spindles during the metaphase. To assess whether TH588 interferes with any of these processes, we investigated centrosome numbers and spindle morphology of mitotic cells in unsynchronized cell cultures. TH588 had no effect on centrosome duplication (Supplementary Fig. S2A) but decreased the separation of duplicated centrosomes in a concentration-dependent manner (Fig. 2A,B and Supplementary Fig. S2B). As a result, cells failed to position their microtubule asters in opposite cell poles and exhibited concentration-dependent degrees of spindle defects and lagging chromosomes. More than 50% of the mitotic cells showed monopolar spindles and uncongressed chromosomes at 4 µM TH588 (Fig. 2B). In contrast, the temporal and spatial localization of aurora kinase A, polo-like kinase 1, and kinesin family member 23 was not altered, suggesting that spindles remained physically intact (Supplementary Fig. S2B).

Live-imaging showed that TH588 trapped cells in metaphase and mitotic duration increased in a concentration-dependent manner (Fig. 2C,D and Supplementary Movies 1–3). Prolonged metaphase was coincidental with lagging chromosomes, suggesting that TH588 activated the spindle checkpoint. The delay was temporary and most cells eventually segregated their chromosomes and exited mitosis. However, at high concentrations of TH588, the majority of cells were permanently arrested in metaphase, or exited mitosis without division (mitotic slippage) (Fig. 2E). Flow cytometry of 5-ethynyl-2-deoxyuridine (EdU)-labeled cells confirmed that TH588 treatment led to accumulation of cells at the G2/M phase of the cell cycle (Fig. 2F,G), in agreement with increased mitotic duration and metaphase arrest.

We next tested the possibility that TH588 targets microtubules or microtubule dynamics by generating cells expressing the microtubule plus-end labeling EB1-GFP reporter (Fig. 2H). Live imaging of single cells before and after drug administration showed that TH588 markedly decreased the mobility of EB1-GFP complexes, and that plus-end immobilization occurred within minutes of drug administration (Fig. 2H and Supplementary Movies 4, 5). Similar experiments with cells expressing a tubulin-GFP fusion protein showed that TH588 reduced microtubule dynamics without altering microtubule filament density (Supplementary Movie 6). We conclude that TH588, by decreasing microtubule dynamics, disrupts assembly of bipolar spindles and chromosome congression in mitotic cells, thus causing metaphase arrest and prolonged mitoses.

### TH588 disrupts mitotic spindles independently of MTH1

**Figure 3:**
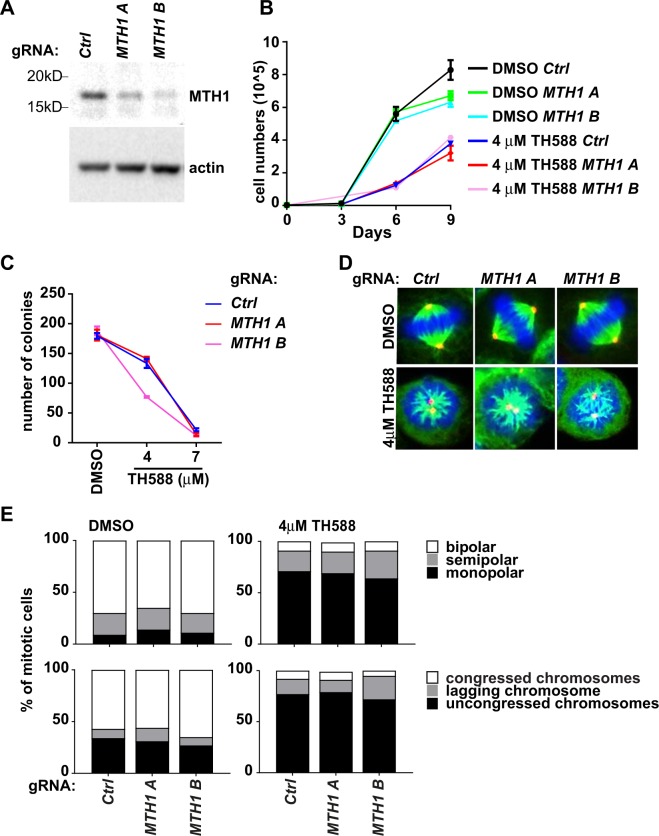
TH588 disrupts mitotic spindles independently of MTH1. (**A**) Western blot of protein extracts from cells infected with a single lentiviral gRNA targeting *MTH1* or non-targeting controls, with antibody against MTH1. Actin was used as loading control. (**B**) Growth curves of DMSO- and TH588-treated *MTH1*-knockout batch clones and control batch clones (n = 4). (**C**) Graph showing colony formation of DMSO- and TH588-treated *MTH1*-knockout batch clones and control batch clones (n = 4). (**D**) Photomicrographs of unsynchronized mitotic cells treated with DMSO or TH588 for 2 hours showing pericentrin (red), α-tubulin (green), and chromatin (blue, DAPI). The cells expressed gRNA targeting *MTH1* or non-targeting control. (**E**) Graphs showing percentages of DMSO- and TH588-treated mitotic cells with bipolar, semipolar or monopolar spindles (top panel), and with congressed, lagging or uncongressed chromosomes (bottom panel). Cells expressed gRNA targeting *MTH1* or non-targeting control (n = 3 replicates/concentration, 100 mitoses/replicate). Graphs show mean +/− SEM.

The mechanism we propose for the cytotoxic effect of TH588 differs from the original proposal, namely inhibition of MTH1-mediated sanitation of the nucleotide pool^1^, but it remains possible that inhibition of MTH1 contributes to the drug effect. To address this, we designed two gRNAs targeting ubiquitous exons in *MTH1* (Supplementary Fig. S3A) and produced *MTH1*-knockout batch clones (Fig. 3A and Supplementary Fig. S4A). Knockout of *MTH1* did not affect growth or colony formation of H460 cells compared to isogenic controls (Fig. 3B,C). Thus, inactivation of *MTH1* did not reproduce the drug effect. Moreover, TH588 reduced the growth and viability of *MTH1*-deficient cells to the same extent as control cells, showing that MTH1 is dispensable for the drug effect. *MTH1* knockout did not affect centrosome separation, formation of bipolar spindles, or chromosome congression in mitotic cells, and *MTH1* knockout did not abolish or attenuate the impact of TH588 on the same features (Fig. 3D,E). Thus, MTH1 inhibition does not contribute to TH588’s effect on mitotic spindles.

### TH588 induces USP28- and p53-dependent G1 arrest

**Figure 4:**
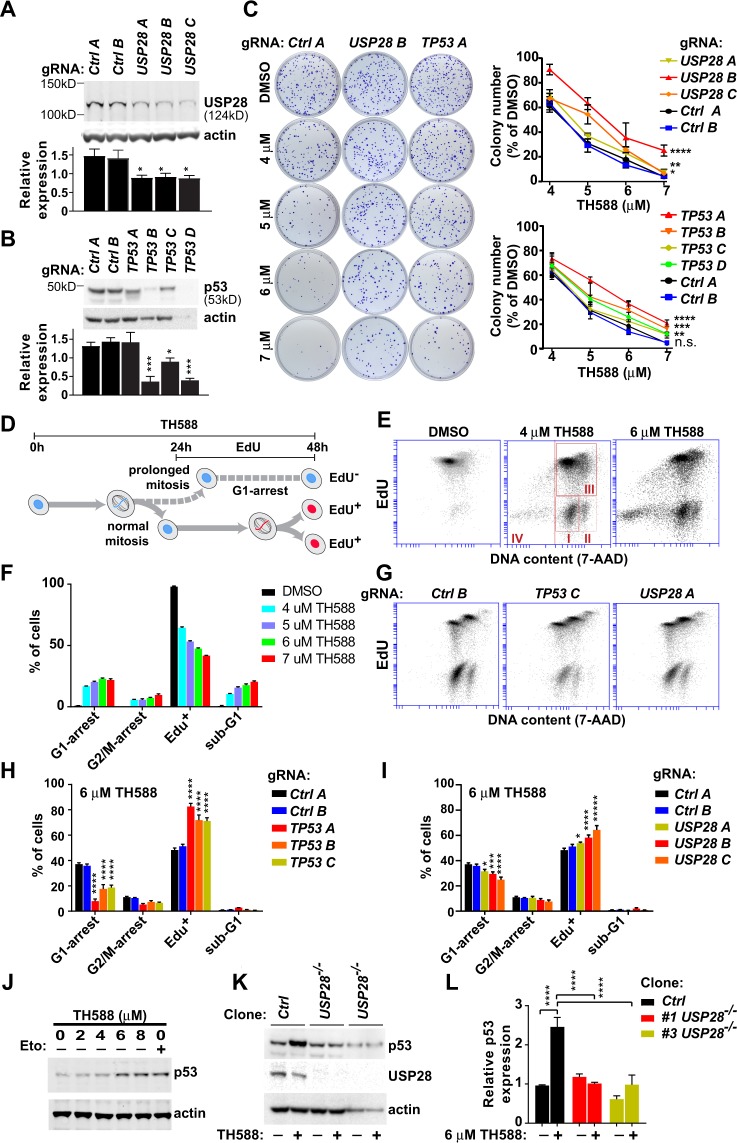
TH588 induces USP28- and p53-dependent G1 arrest. (**A**,**B**) Western blots of protein extracts from cells infected with a single lentiviral gRNA targeting *USP28* (**A**) or *TP53* (**B**) or non-targeting controls, with antibodies against USP28 (**A**) or p53 (**B**). Actin was used as loading control. Graphs showing levels of USP28 or p53 protein normalized against actin (n = 4 batch clones/gRNA). (**C**) Colony formation of batch clones expressing single gRNAs targeting *USP28* or *TP53* or non-targeting controls, treated with DMSO or TH588. Graphs showing colony formation as percent of DMSO treated controls (n = 4 batch clones/gRNA). (**D**) Schematic of the cell cycle analyses performed in panels (E–I): Cells were treated with drug (DMSO or TH588) for 24 hours to ensure that they divided once in the presence of the drug, and with drug plus EdU for another 24 hours to determine whether they re-enter the cell cycle (EdU^+^) or become arrested (EdU^−^). (**E**,**F**) Plots showing DNA content (7AAD) and EdU-incorporation of cells after 48-hour incubation with DMSO or TH588 and 24-hour incubation with EdU. Inserted squares in (**F**) indicate G1-arrested (**I**), G2/M-arrested (II), EdU^+^ (III), and sub-G1 (IV) populations. Graph showing cell cycle distribution (n = 3 replicates/concentration). (**G**,**H,I**) Plots showing DNA content (7-AAD) and EdU-incorporation of *TP53*- and *USP28*-knockout batch clones and non-targeting controls after 48-hour incubation with TH588 (6 µM) and 24-hour incubation with EdU. Cell populations were defined as in (**E**). Graphs showing cell cycle distribution (n = 4 batch clones/gRNA). (**J**) Western blots of protein extracts from cells incubated with DMSO or TH588 for 24 hours, with antibodies against p53. Protein extracts from etoposide (Eto)-treated cells (5 µM) were used as positive control. Actin was used as loading control. (**K**,**L**) Western blots of protein extracts from *USP28*^−/−^ and *USP28*^+/+^ clones treated with DMSO or TH588 for 24 hours, with antibodies against p53 and USP28. Actin was used as loading control. Graph showing levels of p53 normalized against actin (n = 3). Statistics used were one-way ANOVA (**A**,**B**,**L**), two-way ANOVA (**C**), and two-way ANOVA, simple effects within rows (**H**,**I**). *q < 0.05, **q < 0.01, ***q < 0.001, ****q < 0.0001. Graphs show mean +/− SEM.

Three independent CRISPR screens previously identified a USP28-p53 pathway – the mitotic surveillance pathway^17^– that mediates cell cycle arrest after centrosome loss or prolonged mitosis^18–20^. Since TH588 caused prolonged mitoses and *TP53* (the gene encoding p53) and *USP28* was highest ranked in our screen (Fig. 1C), we hypothesized that the TH588-effect on mitotic spindles activates the USP28-p53 pathway. To test this, we infected H460 cells with single lentiviral gRNAs targeting *TP53* or *USP28* and generated knockout batch clones (Fig. 4A,B and Supplementary Figs S3B,C and S4B,C). Cells infected with non-targeting gRNAs were used as negative controls. One gRNA (*TP53 A*) targeting exon-3 of *TP53* failed to reduce the amount of p53, but instead generated a truncated p53 protein that migrated faster in gel electrophoresis (Supplementary Fig. S5). The other gRNAs produced knockout batch clones with varying amounts of contaminating wildtype cells.

We evaluated the batch clones for colony formation at increasing TH588 concentrations. *USP28*-knockout batch clones showed increased colony formation at all drug concentrations compared to controls (Fig. 4C). Similar results were obtained for *TP53*: Three batch clones showed increased colony formation at all concentrations (Fig. 4C), and one showed increased colony formation at the highest concentration (P < 0.01). These results indicate that *USP28* and *TP53* are part of the effector mechanism downstream of TH588.

The aforementioned CRISPR screens showed that USP28 and p53 prevent cells from re-entering the cell cycle after prolonged mitosis by arresting cells at the G1 phase of the next cell cycle^18–20^. To investigate whether TH588 eliminates cancer cells by a similar mechanism, cells were treated with TH588 for 24 hours to ensure that they were exposed to the drug during at least one mitosis, and with TH588 plus EdU for another 24 hours to determine whether the cells enter a second cell cycle (Fig. 4D). TH588 increased the percentage of EdU-negative cells in the G1 phase of the cell cycle, and thus prevented cells from re-entering the cell cycle (Fig. 4E,F). In contrast, more than 98% of the untreated cells were EdU-positive and thus re-entered a second cell cycle. The long exposure to TH588 also induced apoptosis, as evidenced by an expanded sub-G1 population (Fig. 4E,F).

To assess whether p53 and USP28 were involved in the growth arrest, we repeated the experiment with *TP53*- and *USP28*-knockout batch clones. *TP53* deficiency markedly reduced the accumulation of EdU-negative cells in the G1 phase of the cell cycle compared to controls, and increased the proportion of EdU-positive cells (Fig. 4G,H). Similar results were obtained with *USP28*-knockout batch clones, but the effect was weaker (Fig. 4G,I). The weaker effect was likely caused by contamination of wildtype cells that will mask a dependency on *USP28*. We conclude that p53 and USP28 prevent TH588-treated cells from re-entering the cell cycle after exiting prolonged mitoses. Of note, TH588-treated batch clones (knockouts and controls) showed fewer apoptotic cells compared to naive H460 cells, possibly as a result of the insertion of lentivirus into the genome.

Since USP28 is a ubiquitin-specific peptidase that activates p53 by removing ubiquitin in response to centrosome loss or prolonged mitosis^18^, we hypothesized that TH588 activates p53 in a USP28-dependent manner. As expected, TH588 concentration-dependently increased p53 levels with an effect size similar to the DNA-damaging drug etoposide (Fig. 4J and Supplementary Fig. S4D). To address whether USP28 is required for p53 activation, we generated homozygous *USP28*-knockout clones from single cells to circumvent the influence of wildtype or heterozygous cells in the batch clones. Strikingly, there was no difference in p53 levels between untreated and TH588-treated *USP28*^−*/*−^ cells (Fig. 4K,L and Supplementary Fig. S4E). We therefore conclude that USP28 is required for p53 activation by TH588.

## Discussion

We performed an unbiased CRISPR screen to identify knockouts that rescue growth of TH588-treated lung cancer cells. The screen identified a densely connected network of pathways and complexes involved in mitotic spindle regulation, suggesting that TH588 directly or indirectly targets mitotic spindles. In support of this, we show that TH588 concentration-dependently: (1) decreased centrosome separation in mitotic cells, (2) increased the occurrence of monopolar spindles and lagging chromosomes, (3) increased mitotic duration and mitotic slippage, and (4) markedly decreased microtubule dynamics. Importantly, TH588 affected mitotic spindles and microtubule dynamics at the same concentrations that decreased growth and viability of cancer cells in this and other studies^1,3–7^.

While it may seem counterintuitive that inactivation of important regulators of spindle formation promoted growth of TH588-treated cells, positive selection in drug-gene interaction screens could have many underpinnings. Spindle formation is driven by redundant pathways and perturbation of one pathway can be compensated for by the upregulation of others^21,22^. Hence, inactivation of spindle regulators may precondition cells to tolerate TH588 better compared to other cells in the mutant cell pool.

Although we have shown that TH588 is a microtubule-modulating agent, we have not identified its direct target(s). TH588 reduced microtubule dynamics within minutes of drug administration, excluding mechanisms that require transcription, translation, or accumulation of slow-forming metabolites. Moreover, TH588 reduced microtubule dynamics during the interphase of the cell cycle, thus arguing against targets that are confined to the mitotic apparatus, and without disrupting microtubule filaments, suggesting that it may belong to the “kinder and gentler” category of microtubule-modulating agents including noscapinoids^23^. These gentler agents are expected to have less adverse effects than other tubulin-targeting agents.

The mode of action we propose for the cytotoxic effect of TH588 differs from that proposed originally, namely that TH588, by targeting MTH1 and the degradation of oxidized nucleotides, induced replication stress and DNA damage signaling via ataxia telangiectasia mutated (ATM)^1^. Of note, gRNAs targeting *ATM* were not enriched in our screen, and knockout of *MTH1* neither recapitulated nor attenuated the effect of TH588 on cell growth or mitotic spindle assembly. We conclude therefore that targeting MTH1 is not sufficient or required to reduce microtubule dynamics; however, we cannot exclude the possibility that MTH1 inhibition and accumulation of oxidized nucleotides contribute to TH588’s anticancer effect *in vivo*. Indeed, the anticancer effects of other microtubule-modulating agents including vinca alkaloids and taxanes are potentiated by oxidative stress^24,25^.

Our results are consistent with those of an earlier study that investigated alternative mechanisms of TH588 and showed that: (1) TH588-treated cells had a proteomic profile that resembled those generated by tubulin-modulating drugs, (2) TH588 arrested cells in the G2/M phase of the cell cycle, and (3) TH588 inhibited tubulin polymerization in a cell-free system^6^. The latter finding is important because it suggests that TH588 interacts directly with tubulin polymers. Decreased tubulin polymerization required substantially higher concentrations of TH588 (10–30 µM)^6^ than those affecting centrosome separation, spindle assembly, chromosome congression, and mitotic duration in our study (1–2 µM). However, compounds that decrease microtubule polymerization at high concentrations are known to suppress microtubule dynamics at 10–100 fold lower concentrations^26^. Hence, TH588 may interact directly with tubulin polymers.

We also identified a novel effector mechanism that prevents TH588-treated cells from re-entering the cell cycle after prolonged mitosis by arresting cells at the G1 phase of the cell cycle. This mechanism is dependent on USP28 activation of p53 and is supported by our observations showing that: (1) knockout of *USP28* or *TP53* rescued colony formation of TH588-treated cells, (2) long-term TH588 treatment arrested cells at the G1 phase of the cell cycle in a *USP28*- and *TP53*-dependent manner, and (3) knockout of *USP28* abolished p53 activation by TH588.

The effector mechanism is probably identical to the recently identified *P53BP1-USP28-TP53-CDKN1A*-dependent mitotic surveillance pathway that induces G1-arrest after centrosome loss or prolonged mitosis^17–19^, which suggests a role for p53 binding protein-1 (P53BP1) and cyclin-dependent kinase inhibitor-1A (CDKN1A, p21) in the TH588 effector mechanism. In support of this, we observed that TH588 concentration-dependently increased the amount of CDKN1A in H460 cells (data not shown), and gRNAs targeting *CDKN1A* ranked high in our screen (but did not reach significance). *P53BP1* was not targeted in the screen, but previous data show that TH588 increases the number of P53BP1 foci in U2OS cells^1^. We therefore propose that the mechanism underlying the cytotoxic effect of TH588 involves P53BP1 and CDKN1A, but this possibility remains to be tested.

Our observation that TH588 arrested cells at the G1 phase contrasts with an earlier publication showing that TH588 arrested cells at the G2 phase of the cell cycle^6^. However, live imaging in our study revealed that TH588 prolonged mitosis by arresting cells at the metaphase, in agreement with the previously published data^6^, but also showed that the majority of the arrested cells eventually exited mitosis. A substantial proportion of these cells were arrested at the G1 phase of the cell cycle and thus were prevented from re-entering the cell cycle. Thus, the major growth-limiting mechanism of TH588 is p53-dependent G1 arrest. However, sustained exposure to TH588 and stalled mitosis may lead to other irremediable outcomes that do not require p53. In line with this, TH588 is toxic to several cancer cell lines with validated p53 mutations^1^.

One weakness of this study is the use of gRNA libraries that targeted cell cycle and kinase genes only. We might have identified additional mechanisms by using genome-wide libraries. However, the concentration-dependent effect on mitotic spindles, instant reduction of microtubule dynamics, and prompt rescue by *USP28* or *TP53* knockout suggest that we have identified the main mechanism of action responsible for the anticancer effect of TH588. Future studies are required to determine if there are additional mechanisms involved and to define the molecular target of TH588.

Microtubule-modulating agents have been the cornerstone of cancer treatment for more than 50 years^27^. However, the manufacture of these often natural or semi-synthetic compounds is laborious and expensive, and their use is restricted by adverse effects and resistance development. Novel synthetic microtubule-modulating agents, such as TH588, which is well tolerated in mice, are therefore highly requested and expected to complement existing chemotherapies. Controversies regarding the validity of MTH1 as a cancer drug target should not stand in the way of future clinical development of TH588 or its structural homologs.

In summary, we performed an unbiased CRISPR screen to identify pharmacological mechanisms of the MTH1-inhibitor TH588. We found that TH588 is a novel microtubule-modulating agent that attenuates mitotic spindle assembly and eliminates cancer cells by the mitotic surveillance pathway. The mechanism is distinct from the original concept in which TH588 caused replication stress by targeting MTH1 and the degradation of oxidized nucleotides. The result opens up for continued clinical development of TH588 that shows promising results in preclinical cancer studies.

## Materials and Methods

### Cell culture

H460 cells are derived from a large-cell carcinoma, harbor somatic mutations or copy number alterations in *KRAS*, *PI3KCA*, *STK11*, *KEAP1*, and *CDKN2A*, and show intermediate sensitivity towards TH588 (https://cancer.sanger.ac.uk/cell_lines). H460 cells were cultured in Dulbecco’s modified eagle’s medium (DMEM) supplemented with 10% inactivated fetal bovine serum (FBS), 2 mM glutamine, 5 mM non-essential amino acids, and penicillin/streptomycin (100 U/ml). HEK293T cells were cultured in DMEM supplemented with 4 mM glutamine and 10% FBS. The cells were obtained from the American Type Culture Collection (ATCC).

### Generation of doxycycline-inducible FLAG-Cas9-expressing cells

Doxycycline-inducible FLAG-Cas9-expressing cells (dox-FLAG-Cas9) were generated from H460 cells infected with lentivirus carrying the pCW-Cas9 vector (packaged by Cyagene) and cultured for 3 days in puromycin. Clones were generated from single cells and tested for FLAG-Cas9 expression by western blotting of protein extracts from cells incubated with 1 µg/ml doxycycline, with antibodies against FLAG. pCW-Cas9 was a kind gift from David Sabatini & Eric Lander (Addgene # 50661)^28^.

### Production of lentiviral human CRISPR knockout pooled gRNA libraries

The human CRISPR enriched pooled library was a gift from David Sabatini & Eric Lander (Addgene # 51044, 51046, and 51048)^16^. Sub-pools of plasmid libraries enriched for cell cycle and kinase targets were prepared as described^28^. Briefly, plasmid libraries were amplified by transformation in XL-10 gold chemical competent *Escherichia coli* (Stratagene) and harvested using endotoxin-free plasmid maxiprep kit (Qiagen) according to the manufacturer’s protocol. For virus production, HEK293T cells were co-transfected with plasmid libraries and pCMV-dR8.2 (Addgene # 8455) and pCMV-VSV-G vectors (Addgene # 8454) using the Xtreme gene 9 transfection reagent (Roche). Virus-containing culture media was harvested 60 hours after transfection, centrifuged at 4700 g for 15 minutes at 4 °C, passed through 0.45 µm filters to remove cell debris, and stored in aliquots at −80 °C. The virus titers were determined as in^28^.

### Pooled lentiviral screen

Dox-FLAG-Cas9 cells were transduced with lentivirus libraries at a titer producing 50% infected cells, and cultured with blasticidine (5 µg/ml) and doxycycline (1 µg/ml) for 7 days to generate complex mutant cell pools for screening. 15–30 million cells were infected in order to maintain 200-fold coverage with respect to the number of gRNAs in the libraries (10,257 gRNAs targeting 1000 cell cycle genes and 5070 gRNAs targeting 500 kinases). The mutant cell pools were passaged every third day in medium containing: (1) 4 µM TH588, (2) 2 µM auranofin, (3) DMSO, or (4) without drugs or DMSO until the untreated control cells had divided 14 times. The 200-fold coverage was maintained at all stages of the screen.

### Preparation of sequencing libraries

Lentiviral inserts were PCR amplified from genomic DNA using a nested PCR protocol with outer primers targeting the virus backbone^16^ and custom inner primers with binding sequences for Illumina Nextera adaptor read1 and read2 attached to the 5′-ends. After product purification using a PCR cleanup kit (Macherey and Nagel), a third PCR using Illumina Nextera indexing primers (N7xx and S5xx) produced the final sequencing libraries that were diluted, pooled, and sequenced on a HiSeq2500 (Illumina) with paired-end 125 bp read length, and v4 sequencing chemistry. The sequencing was done at the SciLife core facility at Uppsala University. The primers are listed in Supplementary Table 1.

### Sequencing data preprocessing and quality control

Sequencing reads were mapped to gRNA sequences and principal component analysis was carried out with MAGeCK-VISPR^29^ version 0.5.3, specifying the parameters “–reverse-complement”, “–sgrna-len 20” and “–trim 73”, otherwise using default settings. Read count histograms were produced with R.

### Statistical tests for gene selection

To find genes that may be subject to either positive or negative selection under TH588 or auranofin treatment, tests for gRNA enrichment or depletion were performed with the maximum likelihood estimation (MLE) algorithm implemented in MAGeCK-VISPR^29^ 0.5.3. This method allows the simultaneous estimation of gene selection for multiple treatments, while also weighing the effect of each gRNA by its estimated efficiency. Parameters used for the MLE algorithm were: “update-efficiency: true”, “trim-5: 73”, “len: 20” and “–permutation-round 1000”, otherwise default settings. Untreated and DMSO-treated samples were both specified as controls in the design matrix.

### Enrichment analyses

To investigate common biological themes among the top ranking genes, overrepresentation analyses were carried out using ConsensusPathDB^30^. For this analysis, ranked gene lists of the cell cycle and kinase libraries were combined, and a combined background of all cell cycle and kinase library genes was used as a reference. Tests were performed for the Gene Ontology^31^ collections “cellular component”, “molecular function” and “biological process”, as well as pathways and protein complexes derived from Reactome^32^, BioCarta, CORUM^33^ and PID^34^. To further investigate putative interactions between proteins encoded by candidate genes, the online STRING database tool^35^ was used.

### Immunofluorescence

Cells were plated onto glass cover slips in 24-well plates then fixed with formalin or methanol and permeabilized with Triton-X100 in PBS. Cover slips were blocked with fetal bovine serum in PBS or bovine serum albumin in PBST (0.1% Tween-20 in PBS) before incubating with primary and secondary antibodies in PBS. Cover slips were mounted onto microscope slides with Fluoroshield Mounting Medium containing DAPI (ab104139, Abcam). Primary antibodies used were anti-α-tubulin (1:2000, T3559, Sigma-Aldrich), anti-pericentrin (1:1500, ab4448, Abcam), anti-aurora kinase A (1:3000, ab13824, Abcam), anti-polo-like kinase 1 (1:100, sc-17783, Santa Cruz), and anti-kinesin family member 23 (1:500, HPA045208, Atlas antibodies). Secondary antibodies used were anti-mouse-488 (1:250, ab150117, abcam), anti-rabbit-594 (1:250, ab150080, abcam), and anti-rabbit-Cy3 (1:250, ab6939, abcam). Fluorescent images were acquired with an Axioplan 2 microscope (Carl Zeiss) and an AxioCam MRm Camera (Carl Zeiss), or an LSM 880 Airyscan microscope (Carl Zeiss).

### Flow cytometry

Cells were incubated with 5 µM 5-ethynyl-2-deoxyuridine (EdU) for 2 or 24 hours and stained with the Click-it EdU Alexa fluor 488 Flow cytometry assay kit (Life Technologies) with addition of 1 µg/ml 7-aminoactinomycin D (7AAD, Lifetechnology, A1310), according to the manufacturer’s protocol. The cell cycle distribution was determined with a FACScan flow cytometer with CellQuest Pro software (version 4.0.2, Becton Dickinson).

### Live imaging of DNA stained cells

Unsynchronized cells were plated at low confluence onto glass bottom cell imaging 24-well plates (Eppendorf) in DMEM lacking indicator dye 24 hours before the experiment started, with addition of SirDNA Hoeshst (1 µM final concentration, Tebu-bio) 16 hours before start, and TH588 (2–8 µM final concentration) or DMSO 2 hours before start. Time lapse image series were generated using a cell^R microscope and scan^R screening station (Olympus), with 10 minute increments between images. Calculations of mitotic duration was done with MatLab, and percentage of metaphase arrested cells with ImageJ.

### Plus-end tracking and GFP-tubulin imaging

Unsynchronized cells were transfected with pGFP-EB1 using Lipofectamine 3000 (Invitrogen) or transduced with GFP-tubulin using the BacMam 2.0 reagent (Thermo Fisher Technology), according to the manufacturers’ instructions, and plated onto MatTek glass bottom dishes in culture medium without indicator dye. Time lapse image series of single cells before and after administration of 4 µM TH588 or DMSO were generated with an LSM 880 Airyscan microscope and the Zen Black 2.3 software (Carl Zeiss), with 2 second increments between images. Plus-end velocities were determined from time-space plots with the multiple kymograph tool and the read velocities from time-space plots macro in Fiji. The pGFP-EB1 was a kind gift from Lynne Cassimeris (Addgene # 17234)^36^.

### Generation of CRISPR/Cas9 knockout batch clones

Double-stranded DNA oligonucleotides (20nt) with gRNA sequences targeting coding exons in *MTH1*, *USP28*, or *TP53* were cloned into the BamH1 site of the Lenticrisprv2 vector, and co-transfected with pCMV-R8.2, and pCMV-VSV-G into HEK293T cells to produce complete Cas9 and gRNA coding lentivirus. Virus-infected H460 cells were cultured in puromycin for 3 days to produce knockout batch clones that were used in experiments. Lenticrisprv2 was a kind gift from Feng Zhang (Addgene # 52961)^37^. gRNA targeting sequences are listed in Supplementary Table 1.

### Western blot

Protein extracts generated by sonication in urea buffer were loaded onto 12% NuPAGE novex tris glycine gels (Invitrogen) and separated by SDS gel electrophoresis, as described^38^. Proteins were transferred onto 0.2 µm nitrocellulose/filter papers (Biorad) and incubated with primary antibodies anti-FLAG (M2, Sigma), anti-USP28 (A300-898A, Bethyl), anti-p53 (DO-1 (sc126), Santa Cruz), anti-MTH1 (clone 11 A3.2, Merck), and anti-actin (A2066, Sigma Aldrich Atlas). Secondary antibodies for luminescence were anti-rabbit-L27A9 (Cell Signalling) and anti-mouse-ab6728 (Abcam), and for fluorescence were IRDye 680RD Donkey Antirabbit 926–68073 (Ll-CDR) and IRDye 680RD Donkey Antimouse 926–68072 (Ll-CDR). Protein bands were detected with the immubilon western chemiluminescenct HRP substrate (Millipore) using a Chemi Doc Touch Imaging system (Bio Rad) or the Li-Cor Odyssey Imager. Images were quantified with ImageJ.

### Clonogenic assays

500 cells were plated in 10 cm dishes and cultured with TH588 or DMSO for 3 days, followed by 12 days’ culture without drugs. Colonies were fixed and stained by incubation in PBS containing 0.05% crystal violet, 1% formaldehyde, and 1% methanol for 20 minutes. The number of colonies was determined with ImageJ. Data are presented as “percent of DMSO” (number of colonies compared to number of colonies in DMSO treated samples) to compensate for different plating efficiency.

### Statistical analysis

Values are mean ± SEM if not otherwise indicated. Statistics were performed with two-tailed Student’s *t*-test for comparisons of two groups; one-way ANOVA for comparisons of multiple groups; two-way ANOVA for multiple groups and concentrations; two-way ANOVA, simple effects within rows for flow cytometry data; and linear regression with test of non-zero slope for dose-response data. Data were adjusted for multiple comparisons by false discovery rates (two-stage linear step-up procedure of Benjamini, Krieger and Yekutieli). *P* or *q* values indicate comparisons between a TH588-treated sample and control. In graphs with more than 1 control, the highest *P* or *q* value is indicated in the figure. The analyses were performed with GraphPad prism. Differences between groups were considered significant when *P* or *q* < 0.05.

## Supplementary information

Supplementary Information

Movie S1

Movie S2

Movie S3

Movie S4

Movie S5

Movie S6

Data file S1

Data file S2

Table S1

## Data Availability

All data generated or analysed during this study are included in this published article (and its Supplementary Information Files).

## References

[1] Gad H (2014). MTH1 inhibition eradicates cancer by preventing sanitation of the dNTP pool. Nature.

[2] Samaranayake Govindi, Huynh Mai, Rai Priyamvada (2017). MTH1 as a Chemotherapeutic Target: The Elephant in the Room. Cancers.

[3] Ikejiri F, Honma Y, Kasukabe T, Urano T, Suzumiya J (2018). TH588, an MTH1 inhibitor, enhances phenethyl isothiocyanate-induced growth inhibition in pancreatic cancer cells. Oncol Lett.

[4] Petrocchi A (2016). Identification of potent and selective MTH1 inhibitors. Bioorg Med Chem Lett.

[5] Kettle JG (2016). Potent and Selective Inhibitors of MTH1 Probe Its Role in Cancer Cell Survival. J Med Chem.

[6] Kawamura T (2016). Proteomic profiling of small-molecule inhibitors reveals dispensability of MTH1 for cancer cell survival. Sci Rep.

[7] Ellermann M (2017). Novel Class of Potent and Cellularly Active Inhibitors Devalidates MTH1 as Broad-Spectrum Cancer Target. ACS Chem Biol.

[8] Narwal M (2018). Crystal Structures and Inhibitor Interactions of Mouse and Dog MTH1 Reveal Species-Specific Differences in Affinity. Biochemistry.

[9] Hashiguchi K, Hayashi M, Sekiguchi M, Umezu K (2018). The roles of human MTH1, MTH2 and MTH3 proteins in maintaining genome stability under oxidative stress. Mutat Res.

[10] Tsuzuki T (2001). Spontaneous tumorigenesis in mice defective in the MTH1 gene encoding 8-oxo-dGTPase. Proc Natl Acad Sci USA.

[11] van der Waals LM (2019). Differential anti-tumour effects of MTH1 inhibitors in patient-derived 3D colorectal cancer cultures. Sci Rep.

[12] Warpman Berglund U (2016). Validation and development of MTH1 inhibitors for treatment of cancer. Ann Oncol.

[13] Wang JY (2016). Reactive Oxygen Species Dictate the Apoptotic Response of Melanoma Cells to TH588. J Invest Dermatol.

[14] Aristizabal Prada ET (2017). The MTH1 inhibitor TH588 demonstrates anti-tumoral effects alone and in combination with everolimus, 5-FU and gamma-irradiation in neuroendocrine tumor cells. PLoS One.

[15] Zhang X (2017). Expression and function of MutT homolog 1 in distinct subtypes of breast cancer. Oncol Lett.

[16] Wang T, Wei JJ, Sabatini DM, Lander ES (2014). Genetic screens in human cells using the CRISPR-Cas9 system. Science.

[17] Lambrus BG, Holland AJ (2017). A New Mode of Mitotic Surveillance. Trends Cell Biol.

[18] Fong, C. S. *et al*. 53BP1 and USP28 mediate p53-dependent cell cycle arrest in response to centrosome loss and prolonged mitosis. *Elife* **5**, 10.7554/eLife.16270 (2016). 10.7554/eLife.16270 PMC4946878 27371829

[19] Lambrus BG (2016). A USP28-53BP1-p53-p21 signaling axis arrests growth after centrosome loss or prolonged mitosis. J Cell Biol.

[20] Meitinger F (2016). 53BP1 and USP28 mediate p53 activation and G1 arrest after centrosome loss or extended mitotic duration. J Cell Biol.

[21] Hayward D, Metz J, Pellacani C, Wakefield JG (2014). Synergy between multiple microtubule-generating pathways confers robustness to centrosome-driven mitotic spindle formation. Dev Cell.

[22] Prosser SL, Pelletier L (2017). Mitotic spindle assembly in animal cells: a fine balancing act. Nat Rev Mol Cell Biol.

[23] Stanton RA, Gernert KM, Nettles JH, Aneja R (2011). Drugs that target dynamic microtubules: a new molecular perspective. Med Res Rev.

[24] Alexandre J, Hu Y, Lu W, Pelicano H, Huang P (2007). Novel action of paclitaxel against cancer cells: bystander effect mediated by reactive oxygen species. Cancer Res.

[25] Chiu WH (2012). Vinca alkaloids cause aberrant ROS-mediated JNK activation, Mcl-1 downregulation, DNA damage, mitochondrial dysfunction, and apoptosis in lung adenocarcinoma cells. Biochem Pharmacol.

[26] Jordan MA, Wilson L (2004). Microtubules as a target for anticancer drugs. Nat Rev Cancer.

[27] Gidding CE, Kellie SJ, Kamps WA, de Graaf SS (1999). Vincristine revisited. Crit Rev Oncol Hematol.

[28] Wang Tim, Lander Eric S., Sabatini David M. (2016). Viral Packaging and Cell Culture for CRISPR-Based Screens. Cold Spring Harbor Protocols.

[29] Li W (2015). Quality control, modeling, and visualization of CRISPR screens with MAGeCK-VISPR. Genome Biol.

[30] Kamburov A (2011). ConsensusPathDB: toward a more complete picture of cell biology. Nucleic Acids Res.

[31] Ashburner M (2000). Gene ontology: tool for the unification of biology. The Gene Ontology Consortium. Nature genetics.

[32] Croft D (2011). Reactome: a database of reactions, pathways and biological processes. Nucleic Acids Res.

[33] Ruepp A (2010). CORUM: the comprehensive resource of mammalian protein complexes–2009. Nucleic Acids Res.

[34] Schaefer CF (2009). PID: the Pathway Interaction Database. Nucleic Acids Res.

[35] Szklarczyk D (2015). STRING v10: protein-protein interaction networks, integrated over the tree of life. Nucleic Acids Res.

[36] Piehl M, Cassimeris L (2003). Organization and dynamics of growing microtubule plus ends during early mitosis. Mol Biol Cell.

[37] Sanjana NE, Shalem O, Zhang F (2014). Improved vectors and genome-wide libraries for CRISPR screening. Nat Methods.

[38] Sayin VI (2014). Antioxidants accelerate lung cancer progression in mice. Sci Transl Med.

