# Supplementary material for: The MTH1 inhibitor TH588 is a microtubule-modulating agent that eliminates cancer cells by activating the mitotic surveillance pathway
Source: Sci Rep. 2019 Oct 11;9:14667. doi: 10.1038/s41598-019-51205-w (PMC6789014; doi:10.1038/s41598-019-51205-w)
Supplement: Supplementary file 1 — Supplementary Information [file 41598_2019_51205_MOESM1_ESM.pdf]

Supplementary Materials

The MTH1 inhibitor TH588 is a microtubule-modulating agent that eliminates cancer cells by activating the mitotic surveillance pathway

Authors: Nadia Gul, Joakim Karlsson, Carolina Tängemo, Sanna Linsefors, Samuel Tuyizere, Rosie Perkins, Chandu Ala, Zhiyuan Zou, Erik Larsson, Martin O. Bergö, and Per Lindahl

Fig. S1. CRISPR/Cas9 screening of TH588-treated cells

Fig. S2. TH588 is a microtubule-modulating agent

Fig. S3. Gene structures and gRNA targeting strategies for *MTH1*, *USP28*, and *TP53*

Fig. S4. Full-length western blot images

Fig. S5. Batch clones infected with gRNA TP53 A produce a truncated p53 protein

Movie S1. SirDNA Hoeshst stained cells incubated with DMSO

Movie S2. SirDNA Hoeshst stained cells incubated with 4 µM TH588

Movie S3. SirDNA Hoeshst stained cells incubated with 8 µM TH588

Movie S4. GFP-EB1 expressing cell, before and after administration of 4 µM TH588

Movie S5. GFP-EB1 expressing cell, before and after administration of DMSO

Movie S6. GFP-tubulin expressing cell, before and after administration of 4 µM TH588

Data file S1. Gene rankings based on MAGeCK MLE gene scores

Data file S2. ConsensusPathDB overrepresentation analysis

Table S1. Primers and gRNA sequences

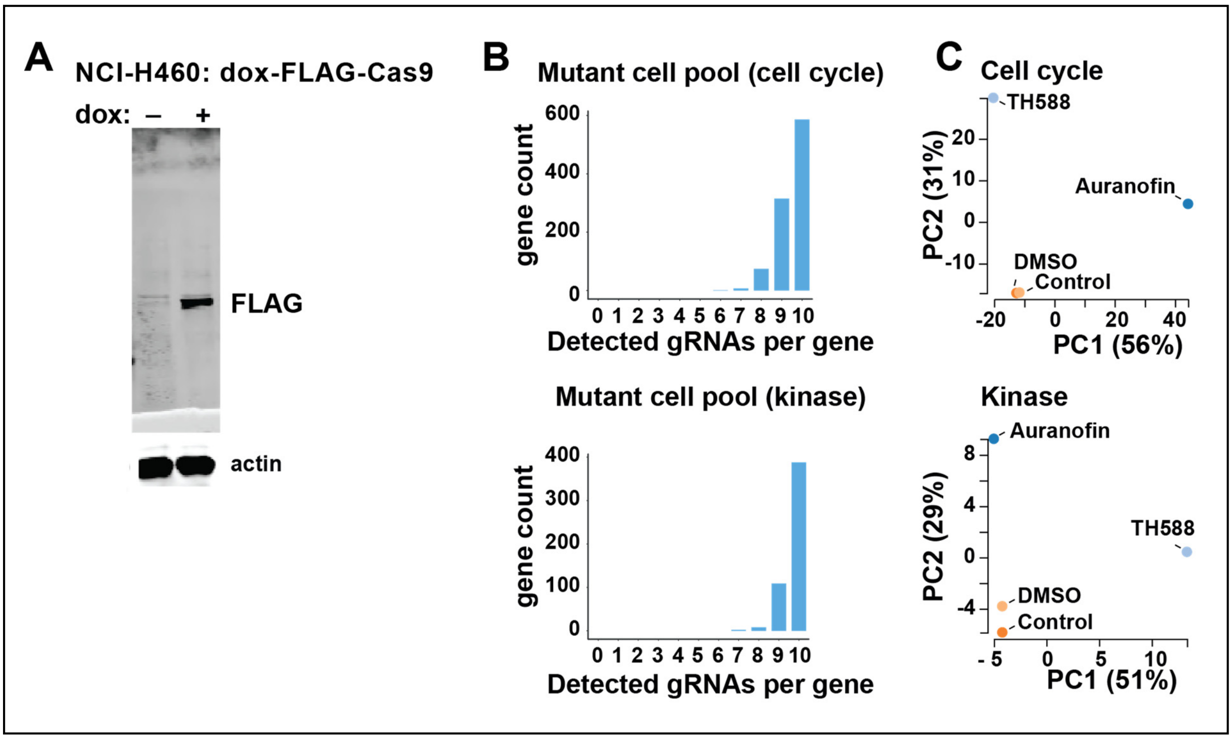

Fig. S1. CRISPR/Cas9 screening of TH588-treated cells. (A) Western blot of protein extracts from a NCI-H460 dox-FLAG-Cas9 clone incubated with or without doxycycline (dox), with antibody against FLAG. Actin was used as loading control. (**B**) Histograms showing the gRNA representation in the MCPs. The number of detected gRNAs (> 20 read counts) per targeted gene is depicted on the x-axis, and the number of target genes for which X number of gRNAs were detected is depicted on the y-axis. (**C**) Principal component analyses of gRNAs targeting cell cycle genes (top) or kinase genes (bottom) showing that TH588- and auranofin- treated samples separated along the two first principal components, in contrast to untreated and DMSO treated control samples that clustered together.

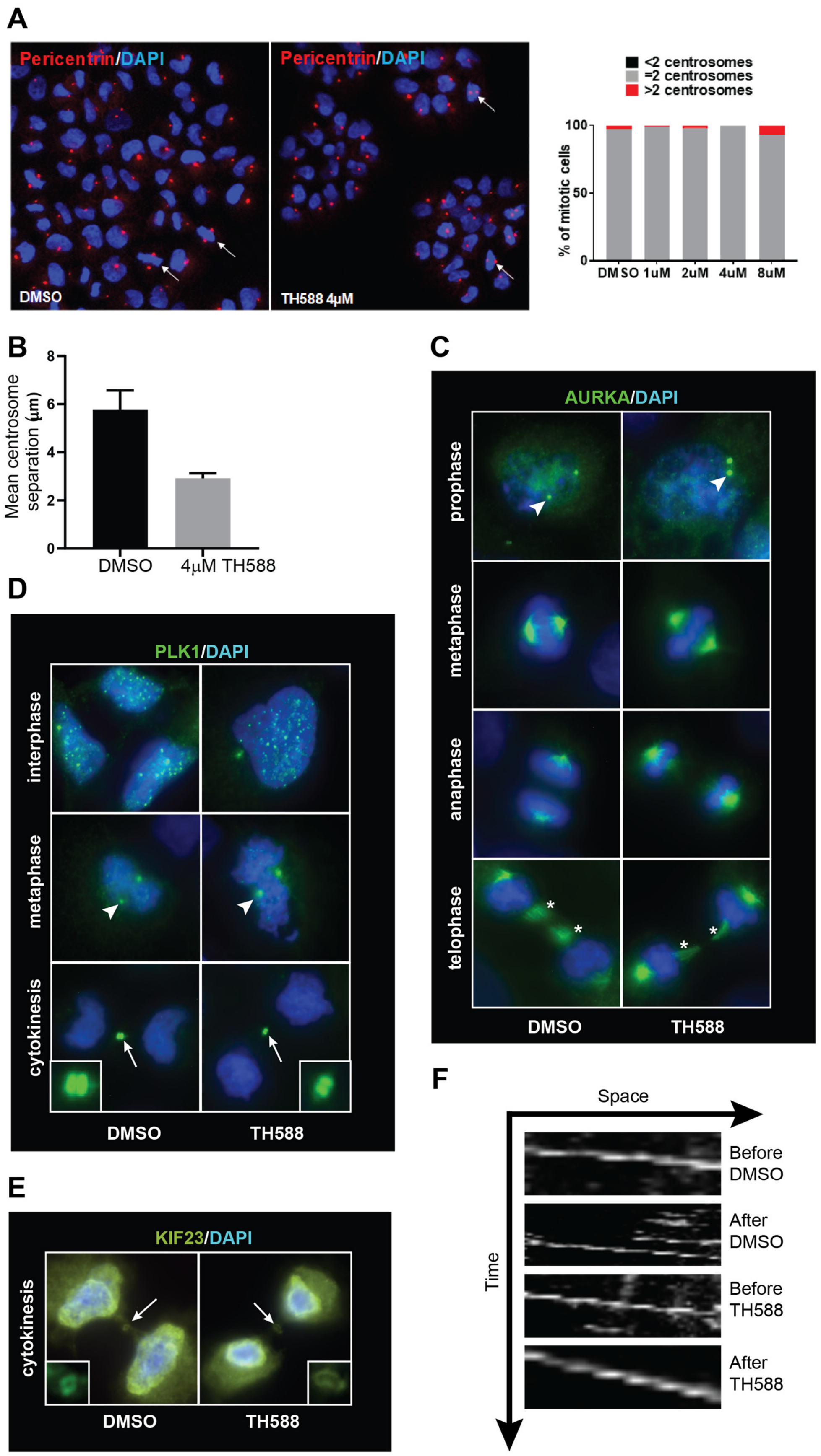
A
Pericentrin/DAPI
DMSO
Pericentrin/DAPI
TH588 4μM
<2 centrosomes
=2 centrosomes
>2 centrosomes
% of mitotic cells
100
50
0
DMSO 1uM 2uM 4uM 8uM
B
Mean centrosome separation (μm)
8
6
4
2
0
DMSO 4μM TH588
C
AURKA/DAPI
prophase
metaphase
anaphase
telophase
DMSO
TH588
D
PLK1/DAPI
interphase
metaphase
cytokinesis
DMSO
TH588
E
KIF23/DAPI
cytokinesis
DMSO
TH588
F
Space
Time
Before DMSO
After DMSO
Before TH588
After TH588

Fig. S2. TH588 is a microtubule-modulating agent. (A) Photomicrographs showing unsynchronized cells treated with DMSO or 4 µM TH588 and stained for pericentrin (red) and DNA (blue, DAPI). Arrows indicate mitotic cells with 2 centrosomes. Graph showing the percentage of mitotic cells treated with DMSO or TH588 (1-8 µM) with < 2 centrosomes, 2 centrosomes, or > 2 centrosomes per cell. (**B**) Graph showing the mean centrosome separation of mitotic cells treated with DMSO or 4 µM TH588, calculated in three dimensions from confocal microscopy images. (**C-E**) Photomicrographs of unsynchronized mitotic cells treated with DMSO or 4 µM TH588 for 2 hours and stained for aurora kinase A (C), polo-like kinase 1 (D), and kinesin family member 23 (E) in green, and DNA in blue (DAPI). Inserts in (D) and (E) show magnified midbodies. Arrowheads indicate centrosomes, asterisks indicate central spindles, and arrows indicate midbodies. (**F**) Kymographs showing representative space-time plots of EB1-GFP plus-ends in interphase cells, before and after administration DMSO or 4 µM TH588.

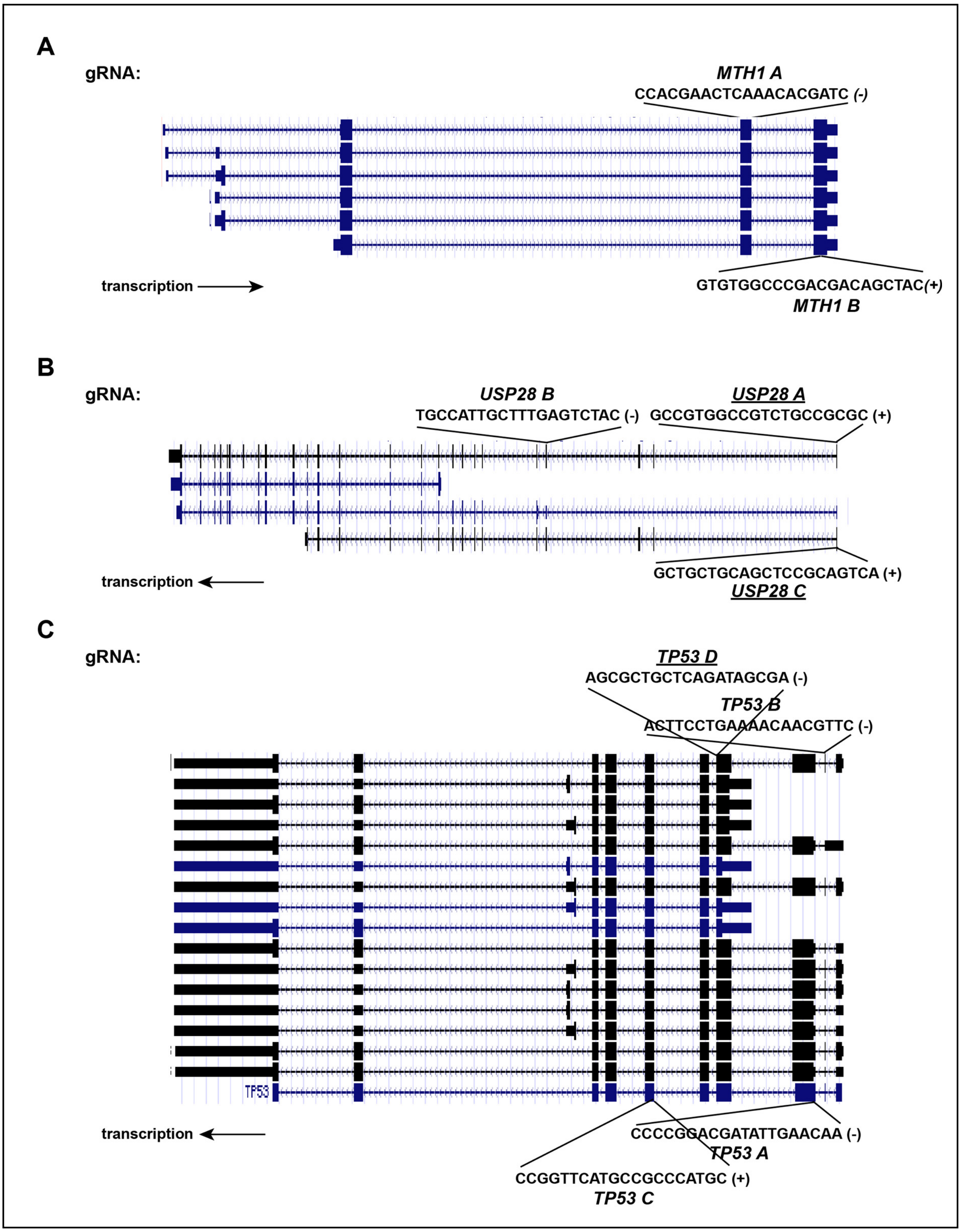

Fig. S3. Gene structures and gRNA targeting strategies for *MTH1*, *USP28*, and *TP53*. (**A-C**) Schematic showing gene structure and splice variants of (A) *MTH1*, (B) *USP28*, and (C) *TP53* extracted from the UCSC genomic browser (http://genome.ucsc.edu/), and the gRNA target sequences used for generating knockout batch clones. The strand orientation of the

protospacer sequences is indicated by + or –. Target sequences that were used in the pooled screen are underlined.

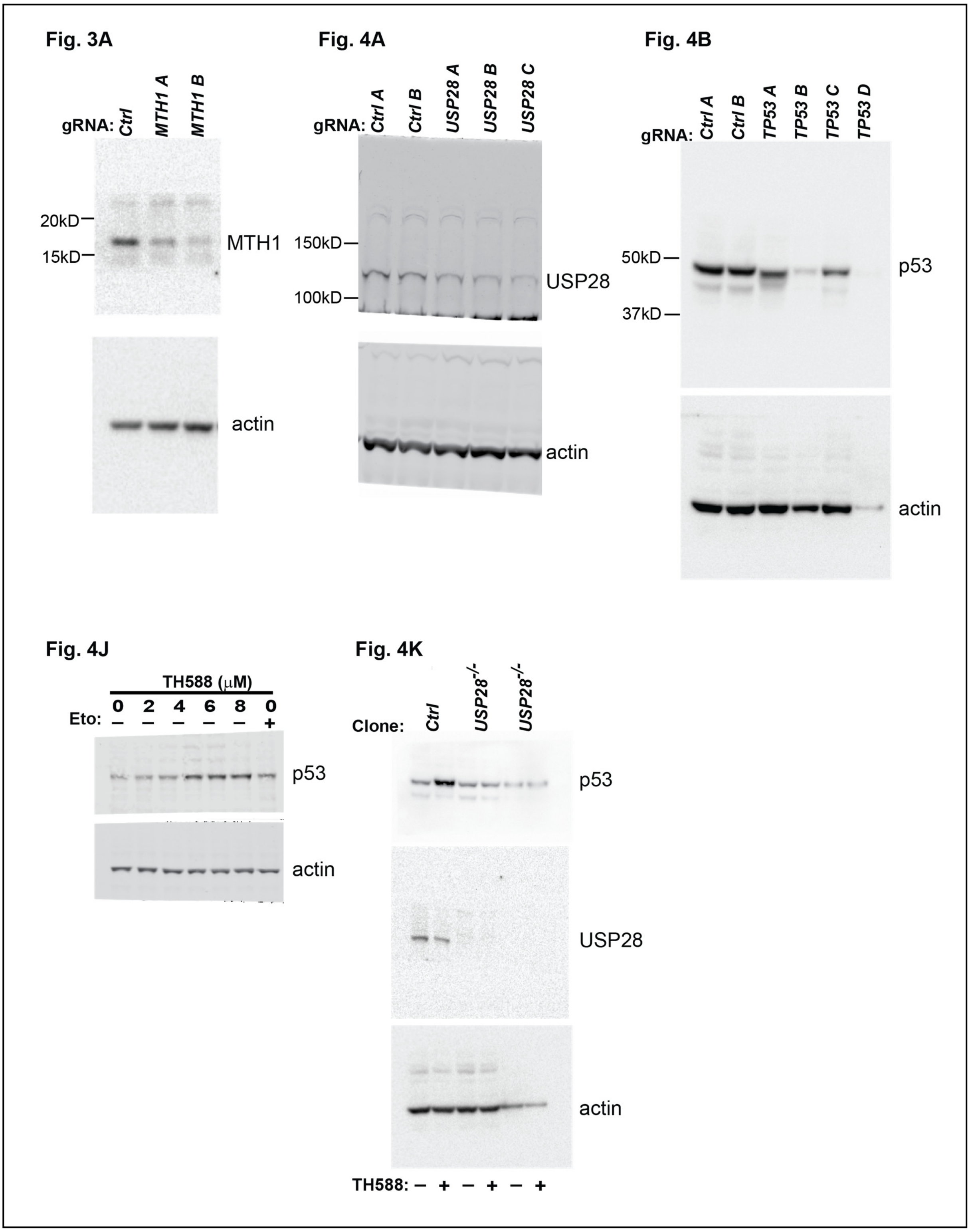

Fig. S4. Full-length western blot images. Some filters were cut before staining to enable detection of multiple antigens.

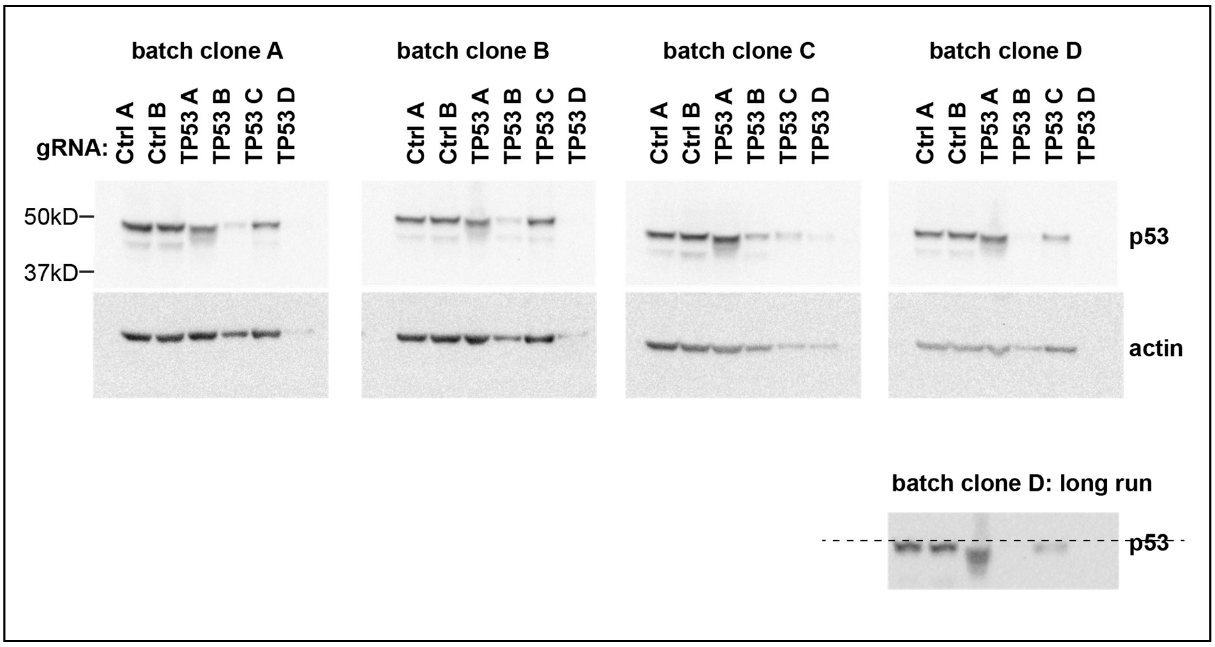

Fig. S5. Batch clones infected with gRNA TP53 A produce a truncated p53 protein. Western blots of protein extracts from 4 independent batch clones of cells infected with 4 different gRNAs targeting TP53 and 2 non-targeting controls, with antibodies against p53. Actin was used as loading control. The lower panel shows batch D after a long electrophoresis run.
